# 

**Published:** 1889-12

**Authors:** 


SUBSCRIBE FOR THE

### International Dental Journal

**The Organ of the Profession,**

PUBLISHED BY THE

INTERNATIONAL DENTAL PUBLICATION COMPANY:

**LOUIS JACK,** *President,* Philadelphia.
**BENJAMIN LORD,** *Vice-President,* New York.
**C. N. PEIRCE,** *Treasurer,* Philadelphia.
**GEO. S. ALLAN,** *Secretary,* 51 W. 37th St., New York.

**W. XAVIER SUDDUTH, M.D., D.D.S., F.R.M.S.**

Editor and Business Manager,

716 Filbert Street, Philadelphia, Pa.

**FOREIGN CORRESPONDENTS:**

Dr. ARKÖVY, Budapesth, Hungary.
W. GEO. BEERS, D.D.S., Ed. Dominion Dental Journal, Montreal.
L. C. BRYAN, D.D.S., Basel.
ALFRED BURNE, D.D.S., Sydney, New South Wales.
F. BUSCH, M.D., Berlin.
GEORGE CUNNINGHAM, Cambridge, Eng.
I. B. DAVENPORT, D.D.S., Paris.
PAUL DUBOIS, Ed. L'Odontologie, Paris.
WILLIAM DUNN, Jr., L.D.S., Florence.
A. H. EDWARDS, D.D.S., Madrid.
W. ST. GEORGE ELLIOTT, M.D., D.D.S., Lon.
OSWALD FERGUS, D.D.S., Glasgow, Scotland.
ALPHONSE FEUCHEL, Zahnarzt, St. Petersburg.
C. R. FLETCHER, D.D.S., Sao Paulo, Brazil.
WALTER HARRISON, L.D.S., D.M.D., Brighton, Eng.
ROBERT S. IVY, D.D.S., Shanghai, China.
N. S. JENKINS, D.D.S., Dresden.
F. KLAPPROTH, Zahnarzt, St. Petersburg.
L. KOLBE, " " "
Dr. GEO. P. E. KUHN, Paris.
W. BOWMAN MACLEOD, L.D.S., Edinburgh.
W. D. MILLER, Ph. D., M.D., D.D.S., Berlin.
WILLIAM SACHS, D.D.S., Breslau.
J. G. VAN MARTER, B.A., D.D.S., Rome.
J. M. WHITNEY, M.D., D.D.S., Honnolula.
LUDWIG WARNEKROS, Zahnarzt, Berlin.
LUDW. ADOLPH WEIL, M.D., Munich.
J. COWAN WOODBURN, M.D., Glasgow.

STOCKHOLDERS:

Geo. S. Allan,
R. R. Andrews,
H. A. Baker,
W. C. Barrett,
A. P. Beale,
S. T. Beale, Jr.,
C. S. Beck,
G. V. Black,
E. A. Bogue,
C. F. W. Bodecker,
W. G. A. Bonwill,
C. A. Brackett,
Edward C. Briggs,
Thos. Bradley,
Albert H. Brockway,
A. E. Brown,
Wm. Carr,
Geo. H. Chance,
G. F. Cheney,
Dwight M. Clapp,
John D. Codman,
Chas. D. Cook,
J. N. Crouse,
Edw. T. Darby,
D. M. Davidson,
Wm. H. Dwinelin,
Chas. J. Essig,
J. N. Farrar,
T. Fillebrown,
W. B. Finney,
T. A. Fletcher,
M. W. Foster,
Chas. E. Francis,
W. F. Fundenberg,
W. H. Fundenberg,
E. C. Fundenberg,
A. H. Gilson,
E. S. Gaylord,
J. P. Geran,
S. H. Guilford,
John I. Hart,
O. E. Hill,
J. F. P. Hodson,
J. Morgan Howe,
Robert Huey,
A. O. Hunt,
Louis Jack,
C. R. Jefferis,
C. A. Kingsbury,
Edw. C. Kirk,
C. R. E. Koch,
W. T. La Roche,
W. K. Leech,
F. A. Levy,
Wilbur F. Litch,
J. Bond Littig,
Benjamin Lord,
Geo. W. Lovejoy, (Canada),
John S. Marshall,
James McManus,
Charles McManus,
E. V. McLeod,
H. J. McKellops,
Dan'l Neal McQuillan,
Horatio C. Meriam,
W. D. Miller, (Berlin),
W. E. Page,
S. B. Palmer,
C. B. Parker,
D. D. Peabody,
S. G. Perry,
C. N. Peirce,
Chas. E. Pike,
W. A. Phreaner,
W. E. Pinkham,
W. H. Potter,
H. C. Register,
Z. T. Sailer,
L. D. Shepard,
Eugene H. Smith,
B. Holly Smith,
H. A. Smith,
S. G. Stevens,
W. X Sudduth,
Ambler Tees,
J. D. Thomas,
James Truman,
Wm. H. Trueman,
F. F. Van Woert,
W. W. Walker,
I. F. Wardwell,
C. S. Wardwell,
G. W. Warren,
T. S. Waters,
Geo. W. Weld,
Julius G. W. Werner,
Jacob L. Williams,
R. B. Winder,
C. A. Woodward,
J. A. Woodward,
W. J. Younger.

SUBSCRIBE FOR THE

### International Dental Journal

**The Organ of the Profession,**

PUBLISHED BY THE

### INTERNATIONAL DENTAL PUBLICATION COMPANY:

**LOUIS JACK,** *President,* Philadelphia.
**BENJAMIN LORD,** *Vice-President,* New York.
**C. N. PEIRCE,** *Treasurer,* Philadelphia.
**GEO. S. ALLAN,** *Secretary,* 51 W. 37th St., New York.

---

### W. XAVIER SUDDUTH, M.D., D.D.S., F.R.M.S.

Editor and Business Manager,

**716 Filbert Street, Philadelphia, Pa.**

---

**FOREIGN CORRESPONDENTS:**

Dr. ARKÖVY, Budapesth, Hungary.
W. GEO. BEERS, D.D.S., Ed. Dominion Dental Journal, Montreal.
L. C. BRYAN, D.D.S., Basel.
ALFRED BURNE, D.D.S., Sydney, New South Wales.
F. BUSCH, M.D., Berlin.
GEORGE CUNNINGHAM, Cambridge, Eng.
I. B. DAVENPORT, D.D.S., Paris.
PAUL DUBOIS, Ed. L'Odontologie, Paris.
WILLIAM DUNN, Jr., L.D.S., Florence.
A. H. EDWARDS, D.D.S., Madrid.
W. ST. GEORGE ELLIOTT, M.D., D.D.S., Lon.
OSWALD FERGUS, D.D.S., Glasgow, Scotland.
ALPHONSE FEUCHEL, Zahnarzt, St. Petersburg.
C. R. FLETCHER, D.D.S., Sao Paulo, Brazil.
WALTER HARRISON, L.D.S., D.M.D., Brighton, Eng.
ROBERT S. IVY, D.D.S., Shanghai, China.
N. S. JENKINS, D.D.S., Dresden.
F. KLAPPROTH, Zahnarzt, St. Petersburg.
L. KOLBE, " " "
Dr. GEO. P. E. KUHN, Paris.
W. BOWMAN MACLEOD, L.D.S., Edinburgh.
W. D. MILLER, Ph. D., M.D., D.D.S., Berlin.
WILLIAM SACHS, D.D.S., Breslau.
J. G. VAN MARTER, B.A., D.D.S., Rome.
J. M. WHITNEY, M.D., D.D.S., Honnolula.
LUDWIG WARNEKROS, Zahnarzt, Berlin.
LUDW. ADOLPH WEIL, M.D., Munich.
J. COWAN WOODBURN, M.D., Glasgow.

---

STOCKHOLDERS:

Geo. S. Allan,
R. R. Andrews,
H. A. Baker,
W. C. Barrett,
A. P. Beale,
S. T. Beale, Jr.,
C. S. Beck,
G. V. Black,
E. A. Bogue,
C. F. W. Bodecker,
W. G. A. Bonwill,
C. A. Brackett,
Edward C. Briggs,
Thos. Bradley,
Albert H. Brockway,
A. E. Brown,
Wm. Carr,
Geo. H. Chance,
G. F. Cheney,
Dwight M. Clapp,
John D. Codman,
Chas. D. Cook,
J. N. Crouse,
Edw. T. Darby,
D. M. Davidson,
Wm. H. Dwinelin,
Chas. J. Essig,
J. N. Farrar,
T. Fillebrown,
W. B. Finney,
T. A. Fletcher,
M. W. Foster,
Chas. E. Francis,
W. F. Fundenberg,
W. H. Fundenberg,
E. C. Fundenberg,
A. H. Gilson,
E. S. Gaylord,
J. P. Geran,
S. H. Guilford,
John I. Hart,
O. E. Hill,
J. F. P. Hodson,
J. Morgan Howe,
Robert Huey,
A. O. Hunt,
Louis Jack,
C. R. Jefferis,
C. A. Kingsbury,
Edw. C. Kirk,
C. R. E. Koch,
W. T. La Roche,
W. K. Leech,
F. A. Levy,
Wilbur F. Litch,
J. Bond Littig,
Benjamin Lord,
Geo. W. Lovejoy, (Canada),
John S. Marshall,
James McManus,
Charles McManus,
E. V. McLeod,
H. J. McKellops,
Dan'l Neal McQuillan,
Horatio C. Meriam,
W. D. Miller, (Berlin),
W. E. Page,
S. B. Palmer,
C. B. Parker,
D. D. Peabody,
S. G. Perry,
C. N. Peirce,
Chas. E. Pike,
W. A. Phieaner,
W. E. Pinkham,
W. H. Potter,
H. C. Register,
Z. T. Sailer,
L. D. Shepard,
Eugene H. Smith,
B. Holly Smith,
H. A. Smith,
S. G. Stevens,
W. X. Sudduth,
Ambler Tees,
J. D. Thomas,
James Truman,
Wm. H. Trueman,
F. F. Van Woert,
W. W. Walker,
I. F. Wardwell,
C. S. Wardwell,
G. W. Warren,
T. S. Waters,
Geo. W. Weld,
Julius G. W. Werner,
Jacob L. Williams,
R. B. Winder,
C. A. Woodward,
J. A. Woodward,
W. J. Younger.

SUBSCRIBE FOR THE

### International Dental Journal

**The Organ of the Profession,**

PUBLISHED BY THE

### INTERNATIONAL DENTAL PUBLICATION COMPANY:

**LOUIS JACK,** *President,* Philadelphia.
**BENJAMIN LORD,** *Vice President,* New York.
**C. N. PEIRCE,** *Treasurer,* Philadelphia.
**GEO. S. ALLAN,** *Secretary,* 51 W. 37th St., New York.

**W. XAVIER SUDDUTH, M.D., D.D.S., F.R.M.S.**

Editor and Business Manager,

716 Filbert Street, Philadelphia, Pa.

**FOREIGN CORRESPONDENTS:**

Dr. ARKÖVY, Budapesth, Hungary.
W. GEO. BEERS, D.D.S., Ed. Dominion Dental Journal, Montreal.
L. C. BRYAN, D.D.S., Basel.
ALFRED BURNE, D.D.S., Sydney, New South Wales.
F. BUSCH, M.D., Berlin.
GEORGE CUNNINGHAM, Cambridge, Eng.
I. B. DAVENPORT, D.D.S., Paris.
PAUL DUBOIS, Ed. L'Odontologie, Paris.
WILLIAM DUNN, Jr., L.D.S., Florence.
A. H. EDWARDS, D.D.S., Madrid.
W. ST. GEORGE ELLIOTT, M.D., D.D.S., Lon.
OSWALD FERGUS, D.D.S., Glasgow, Scotland.
ALPHONSE FEUCHEL, Zahnarzt, St. Petersburg.
C. R. FLETCHER, D.D.S., Sao Paulo, Brazil.
WALTER HARRISON, L.D.S., D.M.D., Brighton, Eng.
ROBERT S. IVY, D.D.S., Shanghai, China.
N. S. JENKINS, D.D.S., Dresden.
F. KLAPPROTH, Zahnarzt, St. Petersburg.
L. KOLBE, " " "
Dr. GEO. P. E. KUHN, Paris.
W. BOWMAN MACLEOD, L.D.S., Edinburgh.
W. D. MILLER, Ph. D., M.D., D.D.S., Berlin.
WILLIAM SACHS, D.D.S., Breslau.
J. G. VAN MARTER, B.A., D.D.S., Rome.
J. M. WHITNEY, M.D., D.D.S., Honnolula.
LUDWIG WARNEKROS, Zahnarzt, Berlin.
LUDW. ADOLPH WEIL, M.D., Munich.
J. COWAN WOODBURN, M.D., Glasgow.

**STOCKHOLDERS:**

Frank Abbott,
Geo. S. Allan,
W. W. Allport,
R. R. Andrews,
H. A. Baker,
A. E. Baldwin,
W. C. Barrett,
A. P. Beale,
S. T. Beale, Jr.,
C. S. Beck,
G. V. Black,
C. F. W. Bodecker,
E. A. Bogue,
W. G. A. Bonwill,
C. A. Brackett,
Thomas Bradley,
Edward C. Briggs,
Albert H. Brockway,
A. E. Brown,
Wm. Carr,
Geo. H. Chance,
G. F. Cheney,
Dwight M. Clapp,
John D. Codman,
Chas. D. Cook,
J. N. Crouse,
Edw. T. Darby,
Wm. H. Dwinelle,
Chas. J. Essig,
J. N. Farrar,
T. Fillebrown,
W. B. Finney,
T. A. Fletcher,
M. W. Foster,
Chas. E. Francis,
W. F. Fundenberg,
W. H. Fundenberg,
E. C. Fundenberg,
E. S. Gaylord,
J. P. Geran,
A. H. Gilson,
J. L. Gish,
S. H. Guilford,
John I. Hart,
O. E. Hill,
J. F. P. Hodson,
J. Morgan Howe,
Robert Huey,
A. O. Hunt,
Louis Jack,
V. H. Jackson,
C. R. Jefferis,
C. A. Kingsbury,
Edw. C. Kirk,
C. R. E. Koch,
W. T. La Roche,
W. K. Leech,
F. A. Levy,
Wilbur F. Litch,
J. Bond Littig,
Benjamin Lord,
Geo. W. Lovejoy (Canada),
John S. Marshall,
H. J. McKellops,
Charles McManus,
James McManus,
Dan'l Neal McQuillan,
Horatio C. Meriam,
W. D. Millar (Berlin),
W. E. Page,
S. B. Palmer,
C. B. Parker,
D. D. Peabody,
C. N. Peirce,
S. G. Perry,
Chas. E. Pike,
W. A. Phreaner,
W. E. Pinkham,
W. H. Potter,
Chas. P. Pruyn,
H. C. Register,
Z. T. Sailer,
L. D. Shepard,
Eugene H. Smith,
B. Holly Smith,
H. A. Smith,
C. Stoddard Smith,
P. T. Smith,
S. G. Stevens,
W. X. Sudduth,
Eugene S. Talbott,
Ambler Tees,
J. D. Thomas,
James Truman,
Wm. H. Trueman,
W. W. Walker,
I. F. Wardwell,
C. S. Wardwell,
G. W. Warren,
T. S. Waters,
Geo. W. Weld,
Julius G. W. Werner,
Jacob L. Williams,
R. B. Winder,
C. A. Woodward,
J. A. Woodward,
W. J. Younger.

